# Supplementary material for: Uterine adenomyosis is an oligoclonal disorder associated with KRAS mutations
Source: Nat Commun. 2019 Dec 19;10:5785. doi: 10.1038/s41467-019-13708-y (PMC6923389; doi:10.1038/s41467-019-13708-y)
Supplement: Supplementary file 1 — Supplementary Information [file 41467_2019_13708_MOESM1_ESM.pdf]

Supplementary Information for

Uterine adenomyosis is an oligoclonal disorder associated with *KRAS* mutations

Satoshi Inoue., Yasushi Hirota., Toshihide Ueno., Yamato Fukui., Emiko Yoshida., Takuo Hayashi., Shinya Kojima., Reina Takeyama., Taiki Hashimoto., Tohru Kiyono., Masako Ikemura., Ayumi Taguchi., Tomoki Tanaka., Yosuke Tanaka., Seiji Sakata., Kengo Takeuchi., Ayako Muraoka., Satoko Osuka., Tsuyoshi Saito., Katsutoshi Oda., Yutaka Osuga., Yasuhisa Terao., Masahito Kawazu and Hiroyuki Mano

Correspondence to Satoshi Inoue,

Division of Cellular Signaling, National Cancer Center Research Institute,

5-1-1 Tsukiji, Chuo-ku, Tokyo 104-0045, Japan

Tel.: +81-3-3547-5201

Fax: +81-3-5565-0727

Email: satoshiinouencc4@gmail.com

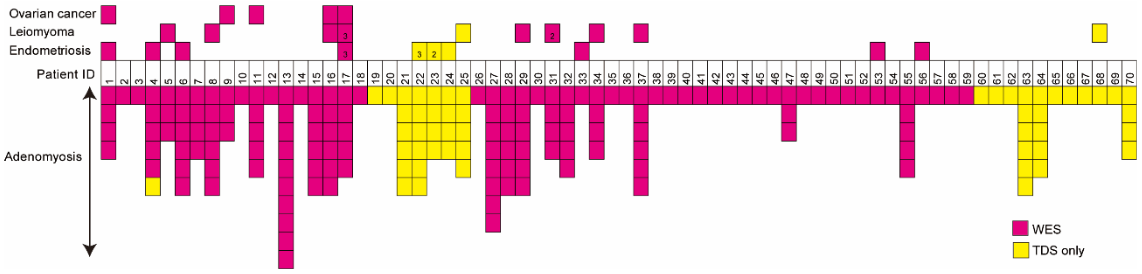

Supplementary Figure 1. Summary of tissue samples sequenced by WES or TDS. Each box represents an individual tissue sample from the indicated patient. In total, 70 adenomyosis patients were profiled, and multiple sites of adenomyosis were sampled for some patients. Co-occurring ovarian cancer, leiomyoma and endometriosis tissues were also profiled for some patients. WES, whole exome sequencing. TDS, targeted deep sequencing.

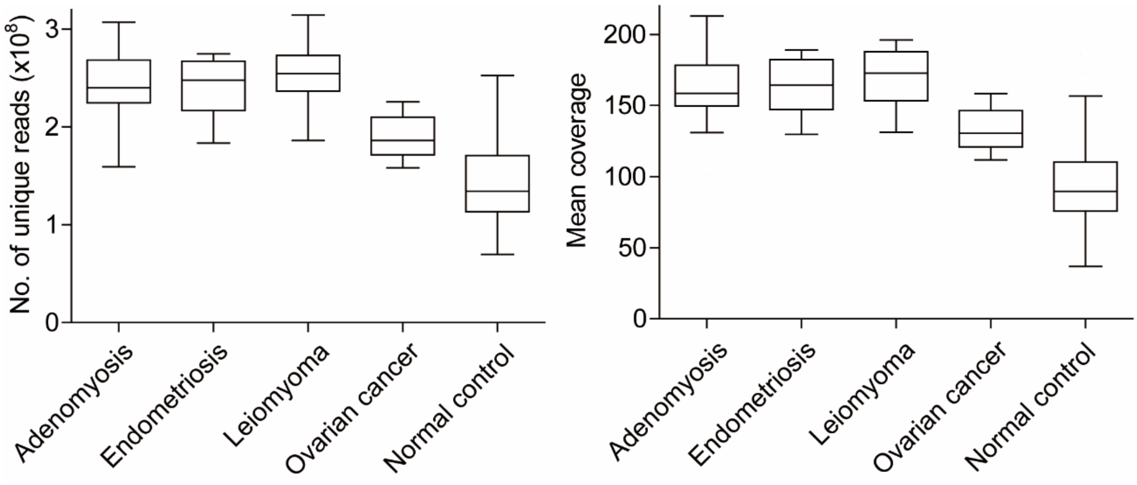

Supplementary Figure 2. Summary of unique reads and mean coverage values of whole exome sequencing (WES).

Distribution of the number of unique reads (left panel) and mean coverage (right panel) for samples subjected to WES, which included 140 adenomyosis, 9 endometriosis, 11 leiomyoma, 5 ovarian cancer, and 51 normal (germline) control samples. The Tukey`s Box-and-Whisker plots were presented.

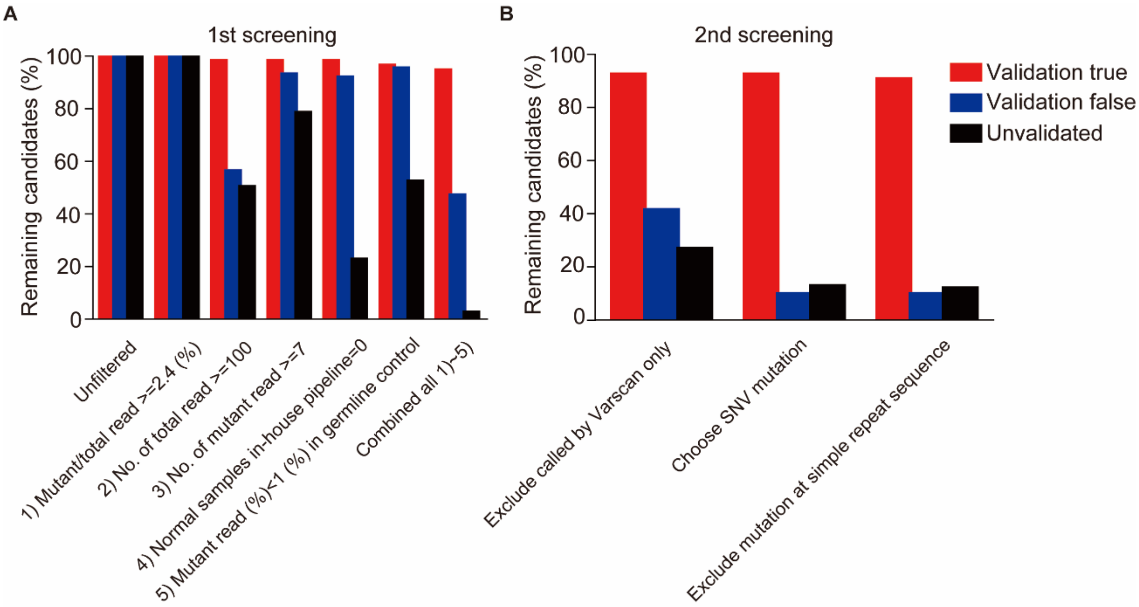

Supplementary Figure 3. Establishment of criteria for calling somatic mutations from whole exome sequencing (WES) data based on targeted deep sequencing (TDS). To define criteria to eliminate false positive mutation calls from WES data made by the Mutect, SID and Varscan variant calling algorithms, TDS of genomic DNA from adenomyosis and normal samples was used to validate WES-identified mutation calls. (A) The percentage of mutation calls remaining after filtering WES-derived variant calls by the indicated criteria. Mutations validated by TDS as true (red bars), false positive mutations (blue bars), and mutations not investigated by TDS (black bars) are shown. Filtering using the 5 indicated parameters eliminated ~50% of false positive mutation calls without significantly affecting true positive mutation calls. (B) Screened mutations calls from (A) were further filtered using the indicated parameters, yielding the indicated percentages of remaining mutations. About 90% of validated false positive and non-validated mutation calls were eliminated without affecting validated mutation calls. This rigorous method for validating WES mutations using TDS identified 7 criteria that improved our ability to make reliable mutation calls.

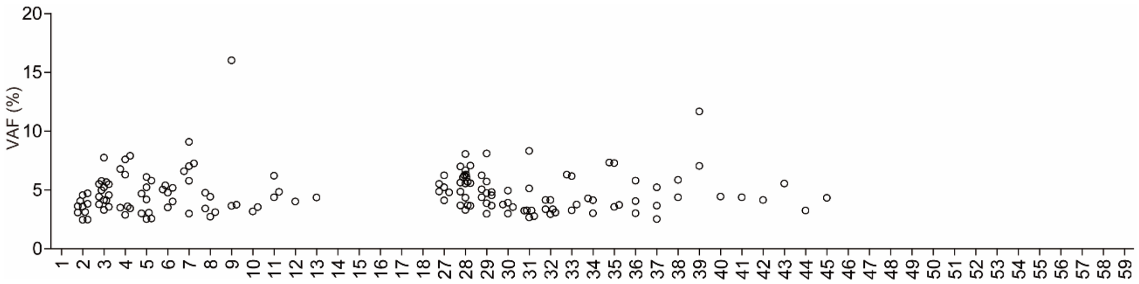

Supplementary Figure 4. Variant allele frequencies (VAF) of single nucleotide variations (SNVs) in adenomyosis as identified by whole exome sequencing (WES). Each circle represents the VAF for an individual SNV detected in the indicated adenomyosis patient.

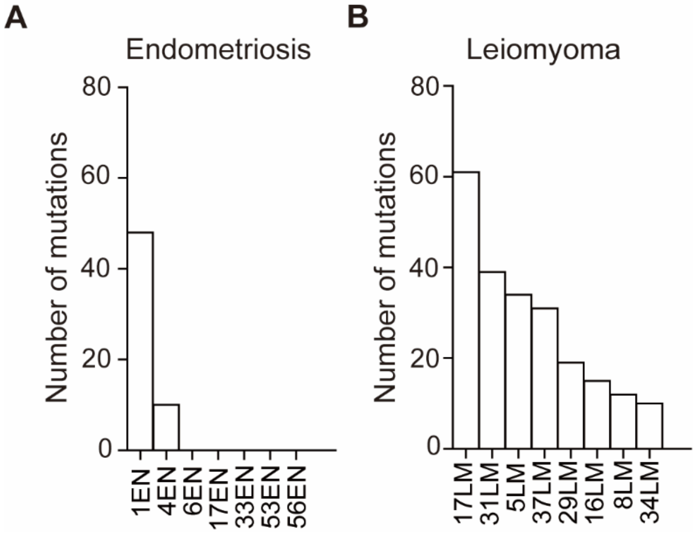

Supplementary Figure 5. Number of mutations identified by whole exome sequencing (WES) in individual endometriosis and leiomyoma samples. Numbers of mutations detected in (A) endometriosis (EN) and (B) leiomyoma (LM) samples from adenomyosis patients with the indicated patient number.

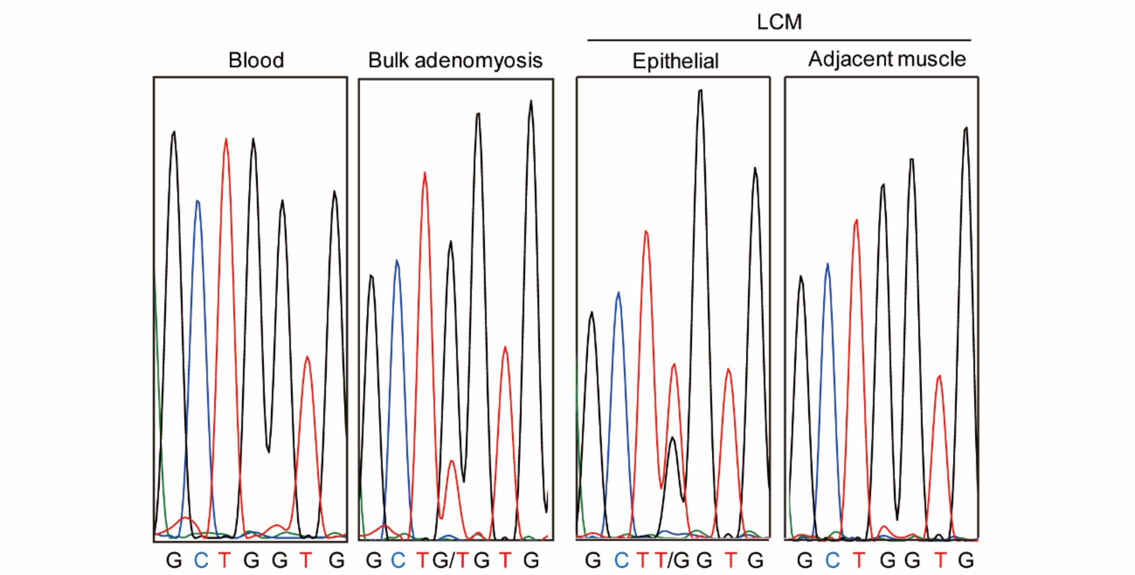

Supplementary Figure 6. Sequencing chromatograms indicating somatic *KRAS* c. 34G>T mutations in the enriched epithelial component of adenomyosis. Genomic DNA sequencing traces for the *KRAS* codon 34 mutation in peripheral blood, a frozen bulk adenomyosis lesion, laser capture micro-dissected (LCM) epithelial component of adenomyosis, or LCM-acquired adjacent muscle tissue from patient #2.

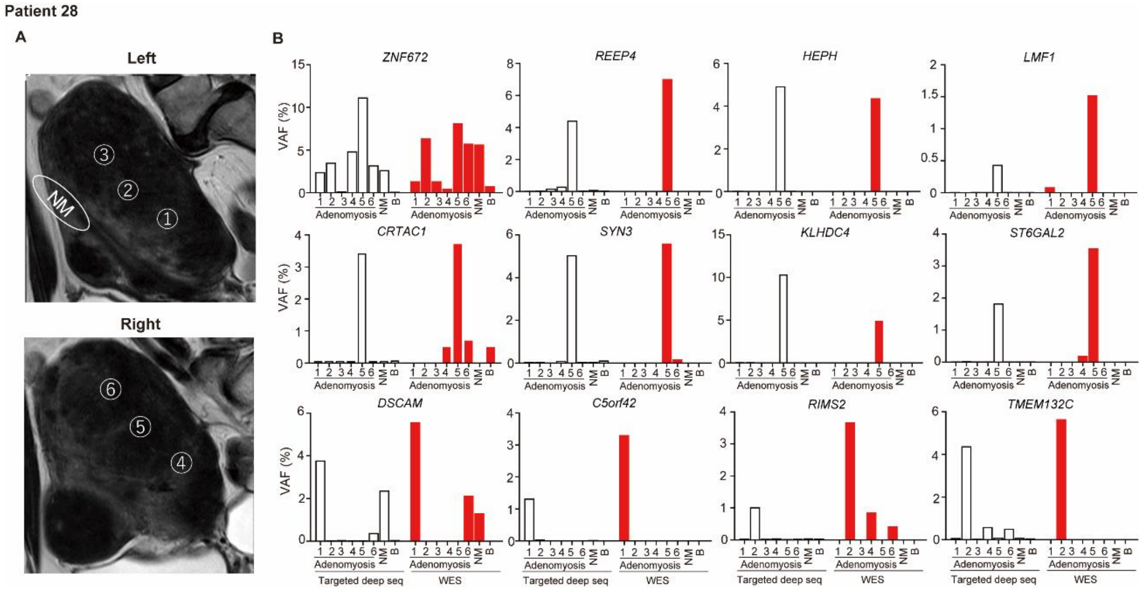

Supplementary Figure 7. Variant allele frequency in multi-regional sampling as determined by targeted deep sequencing (TDS) and whole exome sequencing (WES) for patient #28. (A) Magnetic resonance imaging (MRI) images showing the multi-regional sampling sites of adenomyosis lesions (1~6) and adjacent normal myometrium (NM). (B) VAFs for mutations in the indicated genes across the multi-regional samples in (A) (x-axis) as assessed by TDS (white bars) or WES (red bars). The anatomical positions of these regions are shown in Figure 3A. B, Peripheral blood.

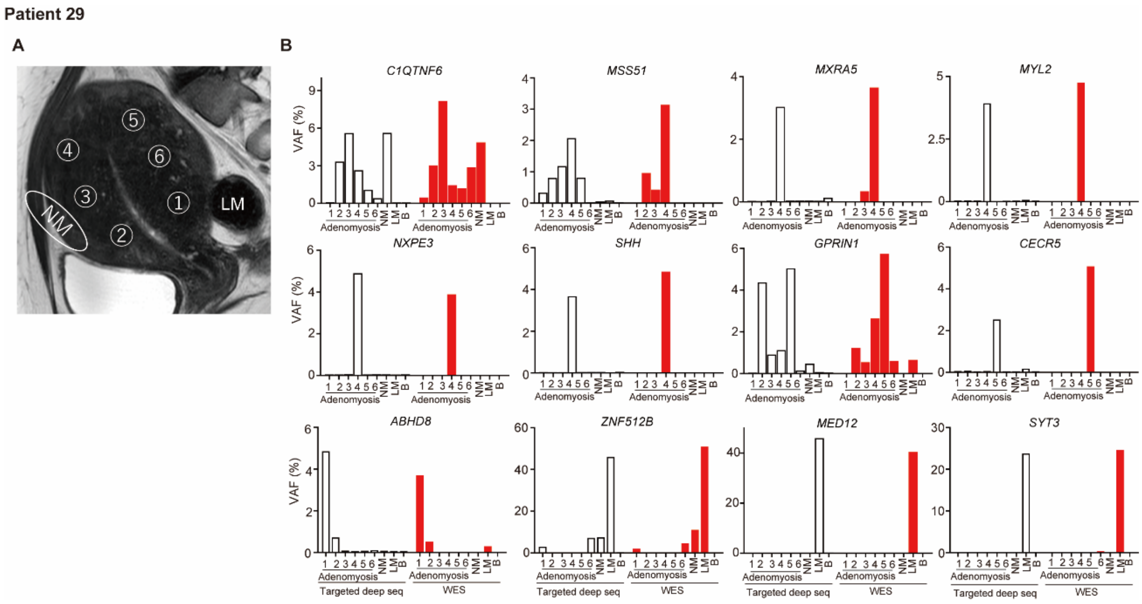

Supplementary Figure 8. Variant allele frequency in multi-regional sampling as determined by targeted deep sequencing (TDS) and whole exome sequencing (WES) for patient #29. (A) Magnetic resonance imaging (MRI) image showing the multi-regional sampling sites of adenomyosis lesions (1−6), adjacent normal myometrium (NM), and a leiomyoma lesion (LM). (B) VAFs for mutations in the indicated genes across multi-regional samples in (A) (x-axis) as assessed by TDS (white bars) or WES (red bars). The anatomical positions of these regions are shown in Figure 3B. B, Peripheral blood.

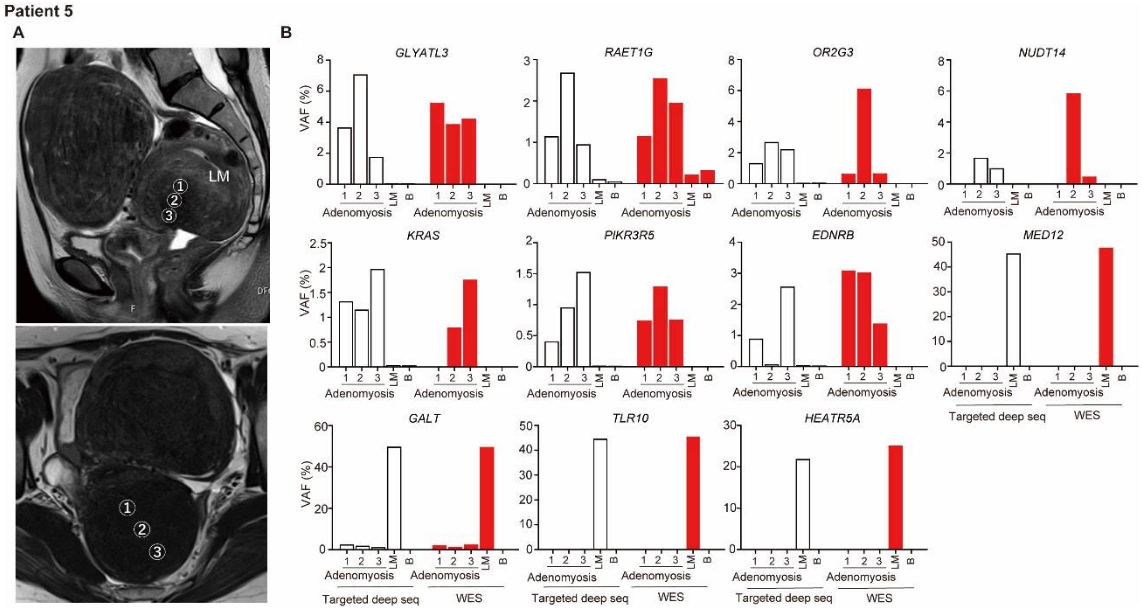

Supplementary Figure 9. Variant allele frequency in multi-regional sampling as determined by targeted deep sequencing (TDS) and whole exome sequencing (WES) for patient #5. (A) Magnetic resonance imaging (MRI) images showing the multi-regional sampling sites of adenomyosis lesions (1−3) and leiomyomas lesions (LM). (B) VAFs for mutations in the indicated genes across multi-regional samples in (A) (x-axis) as assessed by TDS (white bars) or WES (red bars). The anatomical positions of these regions are shown in Figure 3C. B, Peripheral blood.

Patient 8

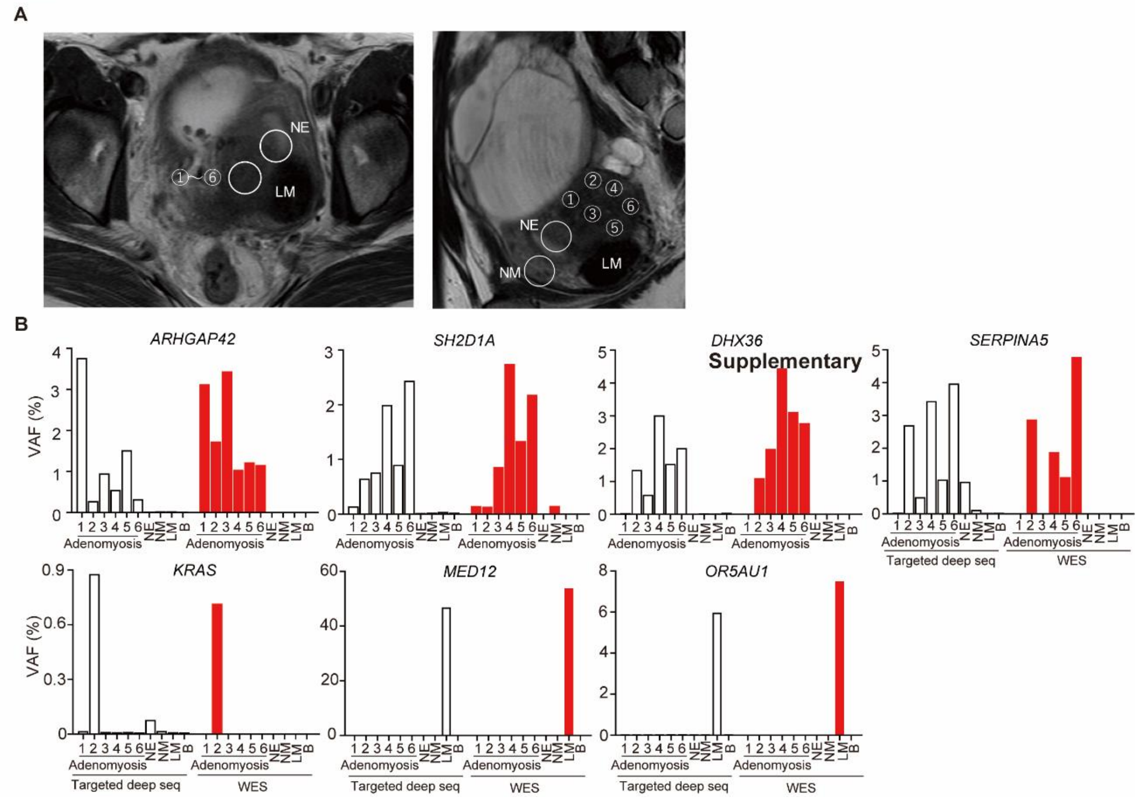

Supplementary Figure 10. Variant allele frequency in multi-regional sampling as determined by targeted deep sequencing (TDS) and whole exome sequencing (WES) for patient #8. (A) Magnetic resonance imaging (MRI) images showing the multi-regional sampling sites of adenomyosis lesions (1−6), adjacent normal endometrium (NE), adjacent myometrium (NM) and leiomyomas (LM). (B) VAFs for mutations in the indicated genes across the multi-regional samples in (A) (x-axis) as assessed by TDS (white bars) or WES (red bars). The anatomical positions of these regions are shown in Figure 4A. B, Peripheral blood.

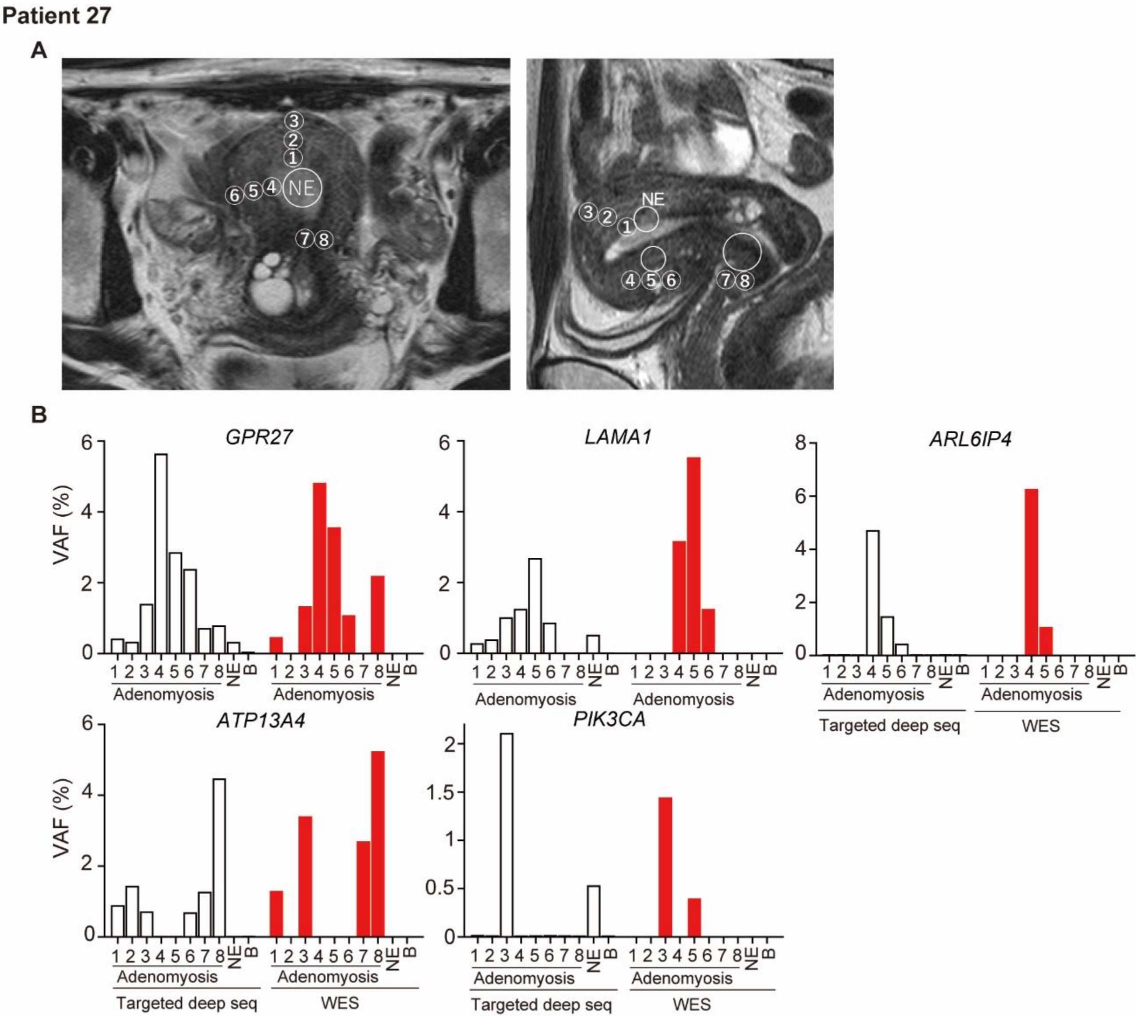

Supplementary Figure 11. Variant allele frequency in multi-regional sampling as determined by targeted deep sequencing (TDS) and whole exome sequencing (WES) for patient #27. (A) Magnetic resonance imaging (MRI) images showing the multi-regional sampling sites of adenomyosis lesions (1−8) and adjacent normal endometrium (NE). (B) VAFs for mutations in the indicated genes across the multi-regional samples in (A) (x-axis) as assessed by TDS (white bars) or WES (red bars). The anatomical positions of regions are shown in Figure 4B. B, Peripheral blood.

Patient 11

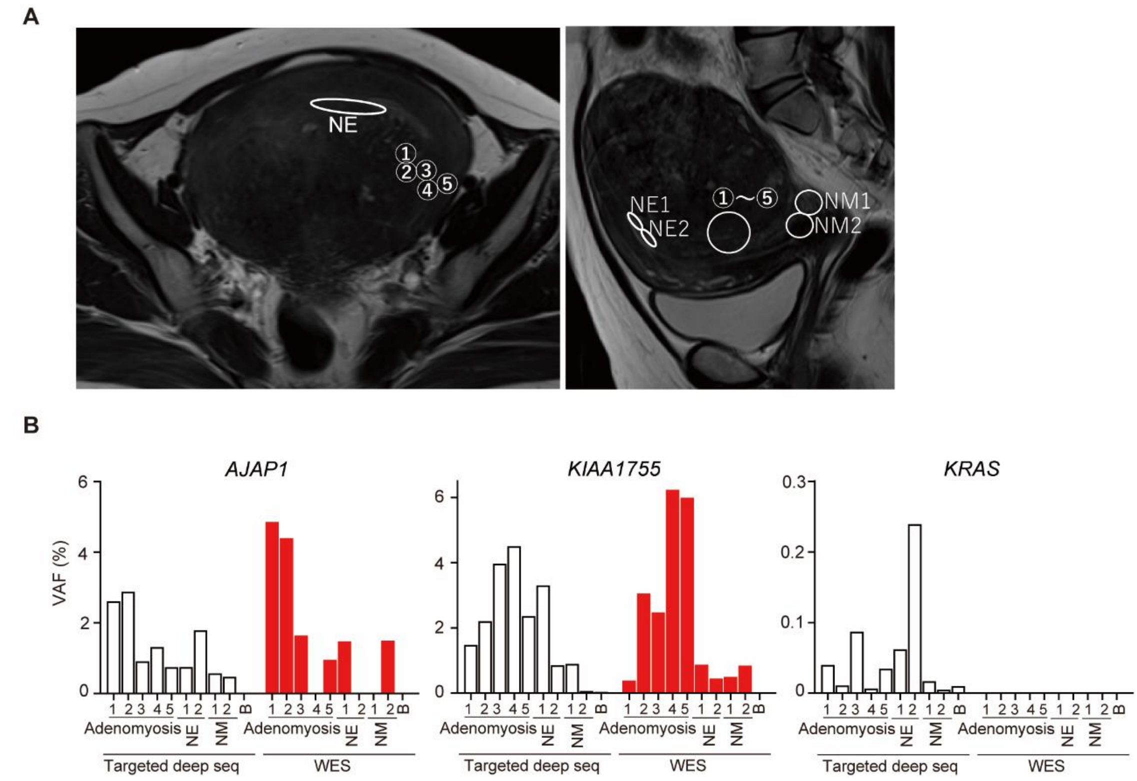

Supplementary Figure 12. Variant allele frequency in multi-regional sampling as determined by targeted deep sequencing (TDS) and whole exome sequencing (WES) for patient #11. (A) Magnetic resonance imaging (MRI) images showing the multi-regional sampling sites of adenomyosis lesions (1−5), adjacent normal myometrium (NM; NM1 and NM2), and adjacent normal endometrium (NE; NE1 and NE2). (B) VAFs for mutations in the indicated genes across the multi-regional samples in (A) (x-axis) as assessed by TDS (white bars) or WES (red bars). The anatomical positions of these regions are shown in Figure 4C. B, Peripheral blood.

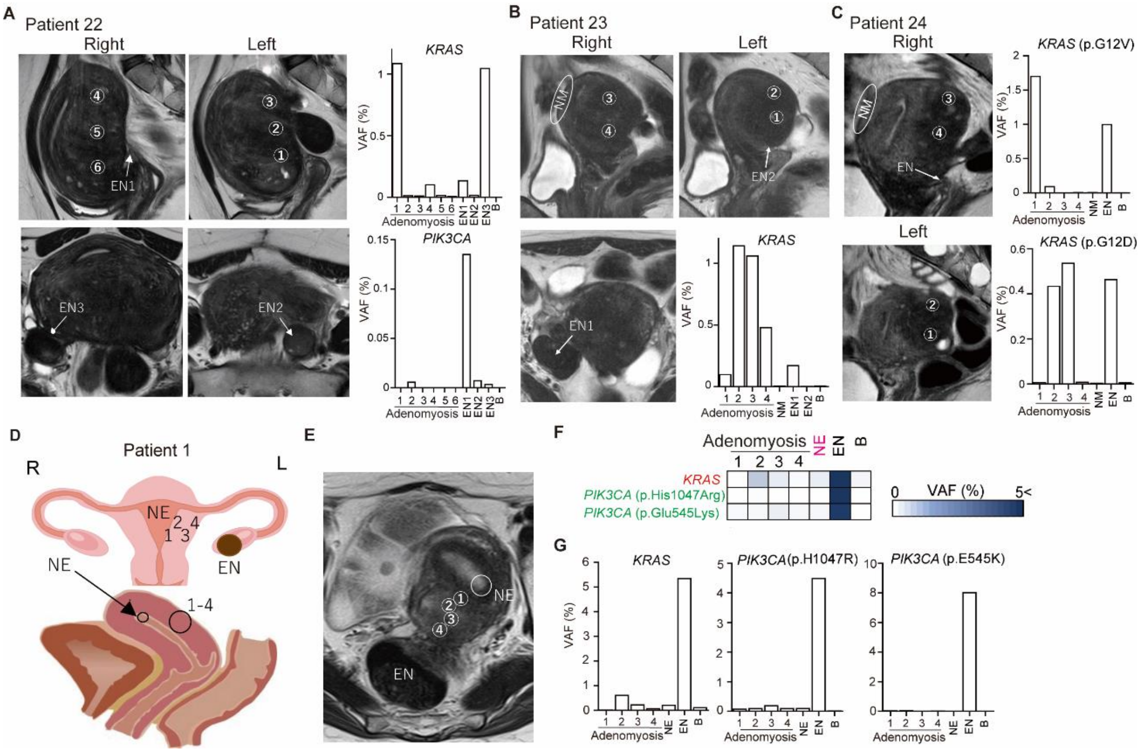

Supplementary Figure 13. Common mutations in adenomyotic and endometriotic lesions as revealed by multi-regional sampling of adenomyosis in patients #22, #23, #24 and #1. (A−C) Left panels: Magnetic resonance imaging (MRI) images showing the multi-regional sampling sites of adenomyosis lesions (1−6 in A, 1−4 in B and C), adjacent myometrium (NM), and endometriosis lesions (EN; EN1−3 in A, EN1−3 in B, EN in C) in the indicated patients. Right panels: VAFs as determined by TDS for mutations in the indicated genes across the multi-regional samples in (A) (x-axis). Peripheral blood (B) was also profiled. Data are related to Figures 4D−F. (D) Coronal plane (top panel) and sagittal plane (bottom panel) schemes for patient 1 showing sites of multi-regional sampling. R, Right; L, Left. (E) Magnetic resonance imaging (MRI) image showing the multi-regional sampling sites of adenomyosis lesions (1−4), adjacent normal endometrium (NE), and endometriosis lesions (EN) in patient #1. (F) VAFs of the indicated mutations in the multi-regional samples in patient 1 shown as a color scale

from dark blue (high frequency) to absence (white). (G) VAFs as determined by TDS for mutations in the indicated genes in the multi-regional samples in (F) (x-axis). Peripheral blood (B) was also profiled.

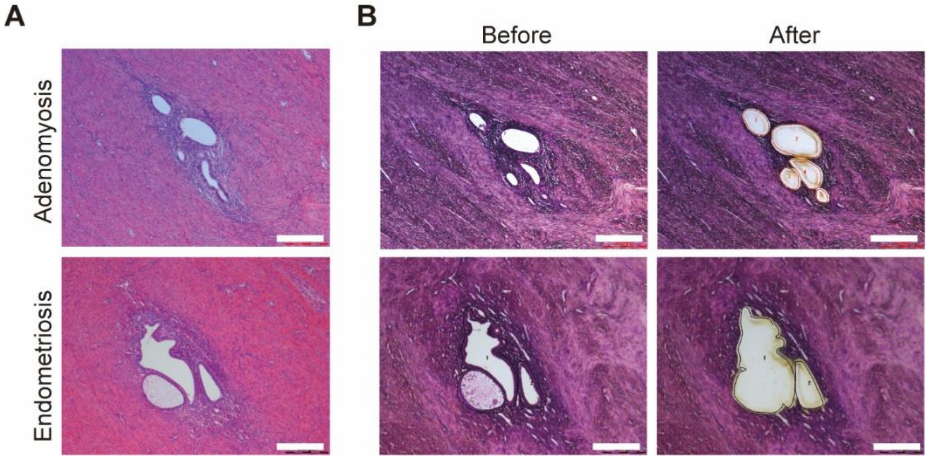

Supplementary Figure 14. Identical *KRAS* mutations in the epithelial components of adenomyotic and endometriotic lesions. (A) Photomicrographs of (upper) adenomyotic and (lower) endometriotic lesions obtained from patient #24 and stained with HE. Scale bar, 100 μm. (B) HE staining of epithelial cells from the (upper) adenomyotic and (lower) endometriotic lesions in (A) before and after isolation by laser capture microdissection (LCM). Scale bars, 100 μm.

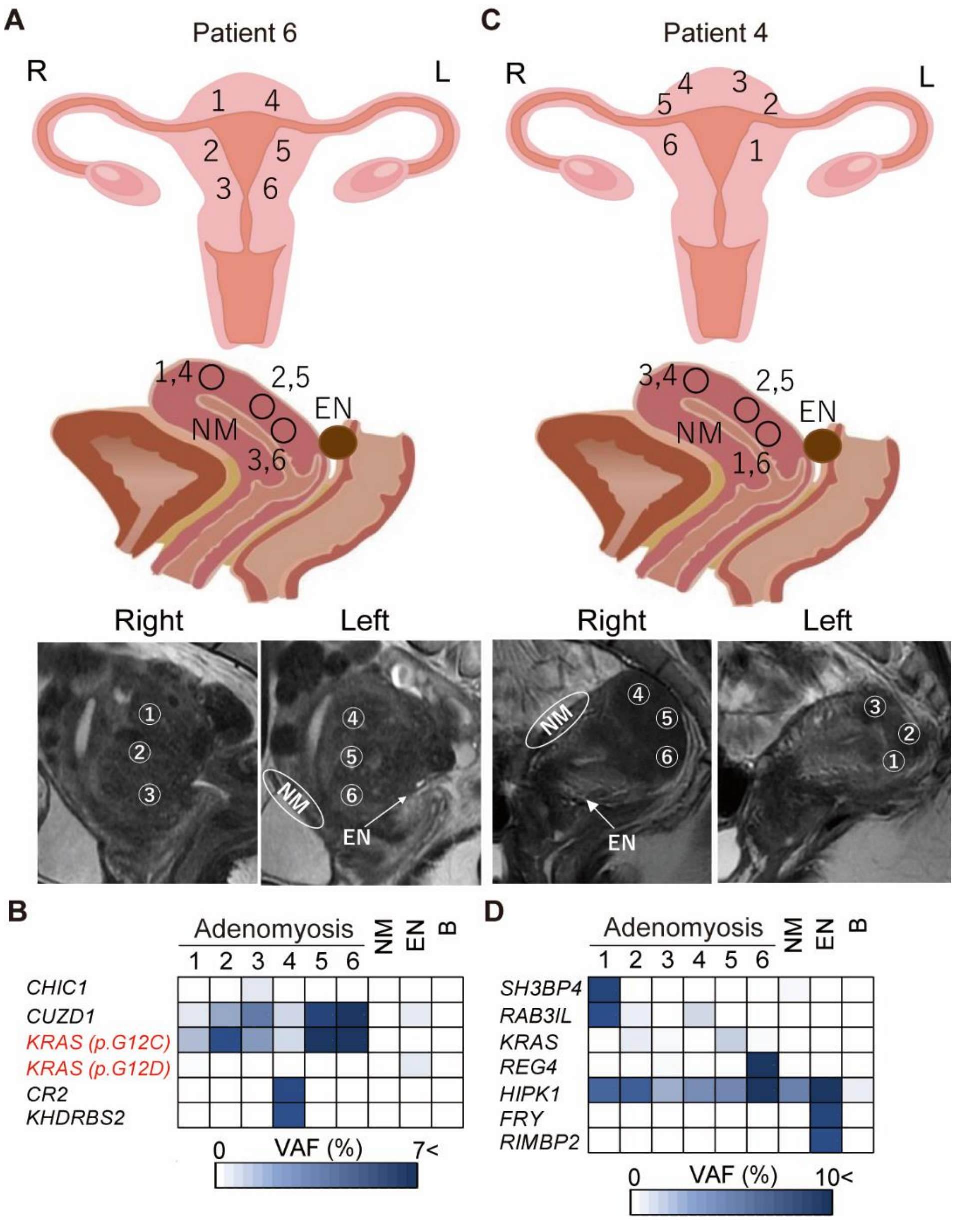

Supplementary Figure 15. Common mutations in adenomyotic and endometriotic lesions as revealed by multi-regional sampling of adenomyosis in patients #6 and #4. (A, C) Coronal plane (top panels) and sagittal plane (middle panels) schemes showing multi-regional sampling sites of adenomyosis lesions (1−6), normal myometrium (NM), and endometriosis (EN) tissues in patient #6 (A) and patient #4 (C). Bottom panels: Magnetic resonance imaging (MRI) images showing the multi-regional sampling sites in the top panels. (B, D) VAFs of the indicated mutations in the

multi-regional samples in (A and C) evaluated as in Figures 4D−F.

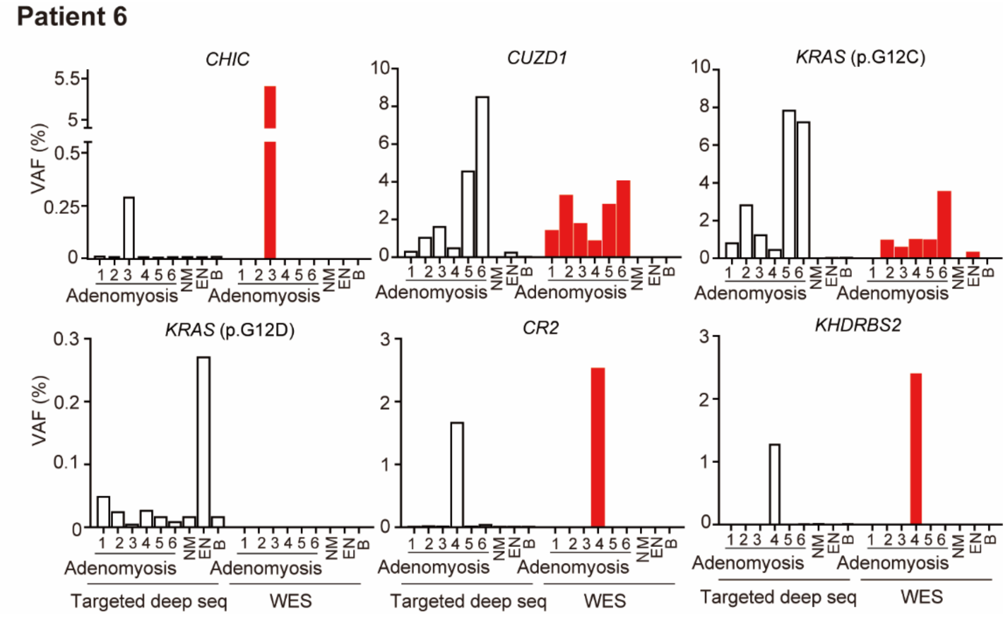

Supplementary Figure 16. Variant allele frequency in multi-regional sampling as determined by targeted deep sequencing (TDS) and whole exome sequencing (WES) for patient #6. VAFs for mutations in the indicated genes across multi-regional samples (x-axis), including 6 adenomyosis lesions and multiple non-diseased tissues, as assessed by TDS (white bars) or WES (red bars). The anatomical positions of these regions are shown in Supplementary Figure 15A. NM, Adjacent normal myometrium; EN, endometriosis; B, Peripheral blood.

Patient 4

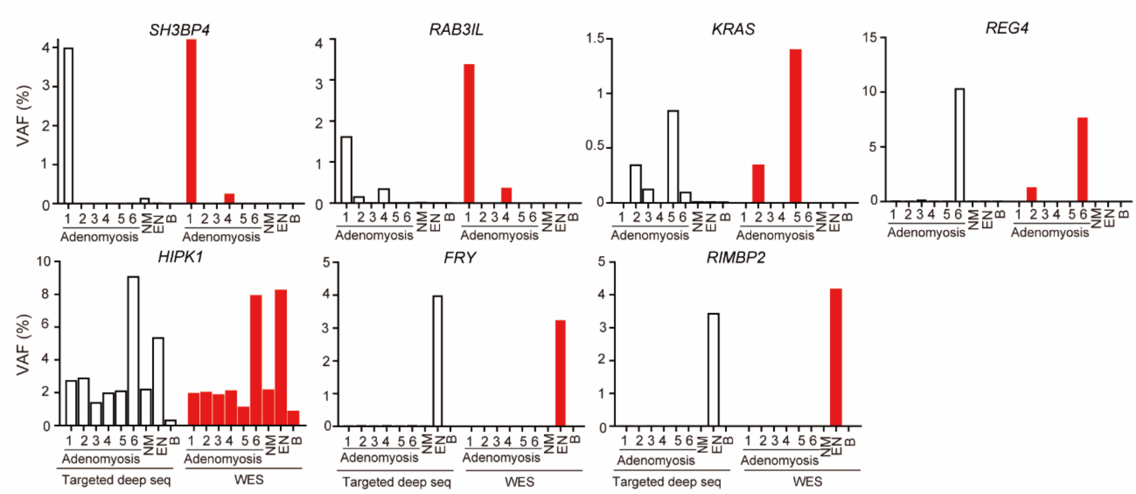

Supplementary Figure 17. Variant allele frequency in multi-regional sampling as determined by targeted deep sequencing (TDS) and whole exome sequencing (WES) for patient #4. VAFs for mutations in the indicated genes across multi-regional samples (x-axis), including 6 adenomyosis lesions and multiple non-diseased tissues, as assessed by TDS (white bars) or WES (red bars). The anatomical positions of these regions are shown in Supplementary Figure 15C. NM, Adjacent normal myometrium; EN, endometriosis; B, Peripheral blood.
