# Supplementary material for: Uterine adenomyosis is an oligoclonal disorder associated with KRAS mutations
Source: Nat Commun. 2019 Dec 19;10:5785. doi: 10.1038/s41467-019-13708-y (PMC6923389; doi:10.1038/s41467-019-13708-y)
Supplement: Supplementary file 3 — Description of Additional Supplementary Files [file 41467_2019_13708_MOESM3_ESM.pdf]

Description of Additional Supplementary Files

File Name: Supplementary Data 1
Description: Clinical characteristics of the adenomyosis patient cohort.

File Name: Supplementary Data 2
Description: Clinical characteristics of patients except for Figure 5.

File Name: Supplementary Data 3
Description: Method used to detect genomic alterations in tissue samples.

File Name: Supplementary Data 4
Description: Read depth of whole exome sequencing for all tissue samples.

File Name: Supplementary Data 5
Description: PCR primers used in this study.

File Name: Supplementary Data 6
Description: SNVs in adenomyosis detected by WES.

File Name: Supplementary Data 7
Description: SNVs in endometriosis detected by WES.

File Name: Supplementary Data 8
Description: SNVs in leiomyoma detected by WES.

File Name: Supplementary Data 9
Description: SNVs in ovarian cancer detected by WES.

File Name: Supplementary Data 10
Description: WES read information for pathogenic COSMIC mutations.

File Name: Supplementary Data 11
Description: Pseudo-positive pathogenic mutations assessed by TDS.

File Name: Supplementary Data 12

Description: Somatic driver mutations as assessed by TDS.

File Name: Supplementary Data 13
Description: Targeted deep sequencing read information of LCM.

File Name: Supplementary Data 14
Description: Multisampling information shown in Figure 3A.

File Name: Supplementary Data 15
Description: Multisampling information shown in Figure 3B.

File Name: Supplementary Data 16
Description: Multisampling information shown in Figure 3C.

File Name: Supplementary Data 17
Description: Multisampling information shown in Figure 4A.

File Name: Supplementary Data 18
Description: Multisampling information shown in Figure 4B.

File Name: Supplementary Data 19
Description: Multisampling information shown in Figure 4C.

File Name: Supplementary Data 20
Description: Multisampling information shown in Figure 4D−F.

File Name: Supplementary Data 21
Description: LCM experiments shown in Figures 4G-I.

File Name: Supplementary Data 22
Description: Patients' characteristics and KRAS mutation related to Table 1.

File Name: Supplementary Data 23
Description: Patients' characteristics and NGS experiments shown in Figure 5.

File Name: Supplementary Data 24

Description: Patients' characteristics and SNV between disease types shown in Figure 5.

File Name: Supplementary Data 25
Description: Patients' characteristics and KRAS mutation status shown in Figure 5.

File Name: Supplementary Data 26
Description: TDS read information shown in Figures 5.

File Name: Supplementary Data 27
Description: Bisulfite sequencing of PR-A/B shown in Figure 8.

File Name: Supplementary Data 28
Description: NGS read information shown in Supplementary Figure 16.

File Name: Supplementary Data 29
Description: NGS read information shown in Supplementary Figure 17.
