# Supplementary material for: Uterine adenomyosis is an oligoclonal disorder associated with KRAS mutations
Source: Nat Commun. 2019 Dec 19;10:5785. doi: 10.1038/s41467-019-13708-y (PMC6923389; doi:10.1038/s41467-019-13708-y)
Supplement: Supplementary file 33 — Reporting Summary [file 41467_2019_13708_MOESM33_ESM.pdf]

nature research

Corresponding author(s): NCOMMS-19-13642C Satoshi Inoue

Last updated by author(s): Nov 18, 2019

Reporting Summary

Nature Research wishes to improve the reproducibility of the work that we publish. This form provides structure for consistency and transparency in reporting. For further information on Nature Research policies, see Authors & Referees and the Editorial Policy Checklist.

Statistics

For all statistical analyses, confirm that the following items are present in the figure legend, table legend, main text, or Methods section.

n/a	Confirmed	
☐	☒	The exact sample size (*n*) for each experimental group/condition, given as a discrete number and unit of measurement
☐	☒	A statement on whether measurements were taken from distinct samples or whether the same sample was measured repeatedly
☐	☒	The statistical test(s) used AND whether they are one- or two-sided<br>*Only common tests should be described solely by name; describe more complex techniques in the Methods section.*
☒	☐	A description of all covariates tested
☒	☐	A description of any assumptions or corrections, such as tests of normality and adjustment for multiple comparisons
☐	☒	A full description of the statistical parameters including central tendency (e.g. means) or other basic estimates (e.g. regression coefficient) AND variation (e.g. standard deviation) or associated estimates of uncertainty (e.g. confidence intervals)
☐	☒	For null hypothesis testing, the test statistic (e.g. *F*, *t*, *r*) with confidence intervals, effect sizes, degrees of freedom and *P* value noted<br>*Give P values as exact values whenever suitable.*
☒	☐	For Bayesian analysis, information on the choice of priors and Markov chain Monte Carlo settings
☒	☐	For hierarchical and complex designs, identification of the appropriate level for tests and full reporting of outcomes
☒	☐	Estimates of effect sizes (e.g. Cohen's *d*, Pearson's *r*), indicating how they were calculated

Our web collection on statistics for biologists contains articles on many of the points above.

Software and code

Policy information about availability of computer code

Data collection	FlowJo software (version 10) for FACs analysis. GIMP (version 2.8) (https://www.gimp.org/) for western blotting image analysis. Bcl2fastq-1.8.4 (Illumina) for WES data collection.
Data analysis	GraphPad Prism (version 5) and Microsoft Excel for most of data analyses. bwa-0.7.12, bowtie2-2.1.0, novocraftV3.02.08.Linux2.6, samtools-0.1.19, bamUtil-1.0.9, GenomeAnalysisTK-2.7-2, snpEff-v4.1b, annovar_2014nov, tabix-0.2.6, muTect-1.1.4, VarScan.v2.3.6, GenomeAnalysisTK-2.3-9 (SomaticIndelDetector) for SNV detection/WES data analyses. Wellcome Trust Sanger Institute mutational signatures framework (ref: Alexandrov LB, Nik-Zainal S, Wedge DC, et al.

For manuscripts utilizing custom algorithms or software that are central to the research but not yet described in published literature, software must be made available to editors/reviewers. We strongly encourage code deposition in a community repository (e.g. GitHub). See the Nature Research guidelines for submitting code & software for further information.

Data

Policy information about availability of data

All manuscripts must include a data availability statement. This statement should provide the following information, where applicable:

- Accession codes, unique identifiers, or web links for publicly available datasets
- A list of figures that have associated raw data
- A description of any restrictions on data availability

WES data are available through the National Bioscience Database Center (ID: JGAS00000000169). All other data are available in the manuscript, Supplementary Methods or Source Data. The source data underlying Figures 5B and 6B-D are provided as a Source Data file.

Field-specific reporting

Please select the one below that is the best fit for your research. If you are not sure, read the appropriate sections before making your selection.

☒ Life sciences ☐ Behavioural & social sciences ☐ Ecological, evolutionary & environmental sciences

For a reference copy of the document with all sections, see nature.com/documents/nr-reporting-summary-flat.pdf

Life sciences study design

All studies must disclose on these points even when the disclosure is negative.

Sample size	No sample-size calculation was performed.
Data exclusions	No data were excluded from the analyses.
Replication	Data reproducibility was examined by six independent experiments per group with three technical replicates within each of the experiments (Figure 6B), four independent experiments per group without technical replicates (Figure 6C), and six independent experiments per group with three technical replicates (three wells/samples) (Figure 6D). Reproducibility of western blotting was confirmed by two independent experiments.
Randomization	Cells were randomly allocated to the various conditions.
Blinding	Scoring of all PR-IHC was performed blind by T.H.Y. to avoid evaluator bias. Investigators were blinded to group allocation during data collection.

Reporting for specific materials, systems and methods

We require information from authors about some types of materials, experimental systems and methods used in many studies. Here, indicate whether each material, system or method listed is relevant to your study. If you are not sure if a list item applies to your research, read the appropriate section before selecting a response.

Materials & experimental systems

n/a	Involved in the study	
☐	☒	Antibodies
☐	☒	Eukaryotic cell lines
☒	☐	Palaeontology
☒	☐	Animals and other organisms
☐	☒	Human research participants
☒	☐	Clinical data

Methods

n/a	Involved in the study	
☒	☐	ChIP-seq
☐	☒	Flow cytometry
☒	☐	MRI-based neuroimaging

Antibodies

Antibodies used	anti-PR(clone: 1E2, Veentana, Cat.No: 790-2223). anti-Vinculin (clone SPM227, abcam, Cat.No: ab18058). anti-HA (clone 6E2, CST, Cat.No: 2367S).
Validation	Specificity of the anti-HA and anti-vinclulin antibodies was validated by overexpression of HA or shRNA of PR. Specificity of the anti-PR antibody was validated by PR-negative patient samples.

Eukaryotic cell lines

Policy information about cell lines

Cell line source(s)	293T: ATCC. Immortalized epithelial endometrial cell was generated by Dr. Muraoka (Nagoya University).
Authentication	None of cell lines were authenticated.
Mycoplasma contamination	All cell lines were tested negative for mycoplasma contamination.
Commonly misidentified lines (See ICLAC register)	No commonly misidentified cell lines were used.

Human research participants

Policy information about studies involving human research participants

Population characteristics	All requested information is provided in Table 1 and Supplementary Tables 1, 2, 22 and 23 in this manuscript.
Recruitment	Patients with adenomyosis gave written informed consent prior to their participation in this study were obtained at the University of Tokyo Hospital and Juntendo University Hospital between December 2016 and July 2019.
Ethics oversight	This project was approved by the institutional ethics committees of the University of Tokyo (Project Number G10035), the Juntendo University Faculty of Medicine (Project Number 2014176), and the National Cancer Center Research Institute (Project Number 2015–202).

Note that full information on the approval of the study protocol must also be provided in the manuscript.

Flow Cytometry

Plots

Confirm that:

- [ ] The axis labels state the marker and fluorochrome used (e.g. CD4-FITC).
- [ ] The axis scales are clearly visible. Include numbers along axes only for bottom left plot of group (a 'group' is an analysis of identical markers).
- [ ] All plots are contour plots with outliers or pseudocolor plots.
- [ ] A numerical value for number of cells or percentage (with statistics) is provided.

Methodology

Sample preparation	Cells were collected 45 min after BrdU exposure, washed by PBS(-), fixed in Cytofix/Cytoperm buffer BD Bioscience; APC-BrdU kit, Cat.No:557892) and permeabilized with Cytofix/Cytoperm buffer (BD Bioscience; APC-BrdU kit, Cat.No:557892). The cells were fixed with Cytofix/Cytoperm buffer (BD Bioscience; APC-BrdU kit, Cat.No:557892), were exposed to DNase BD Bioscience; APC-BrdU kit, Cat.No:557892). The cells were stained with APC-anti-BrdU Ab (BD Bioscience; APC-BrdU kit, Cat.No:557892) for 1h at 4 degrees, followed by 7-AAD (BD Bioscience; APC-BrdU kit, Cat.No:557892) for 10 min.
Instrument	BD FACs Canto II
Software	Flow Jo software (Version 10)
Cell population abundance	No cell sorting was performed.
Gating strategy	Based on FSC vs SSC, cellular debris and cell doublets were excluded.

- [ ] Tick this box to confirm that a figure exemplifying the gating strategy is provided in the Supplementary Information.
